# Assessment of Language and Indexing Biases Among Chinese-Sponsored Randomized Clinical Trials

**DOI:** 10.1001/jamanetworkopen.2020.5894

**Published:** 2020-05-28

**Authors:** Yuanxi Jia, Doudou Huang, Jiajun Wen, Yehua Wang, Lori Rosman, Qingkun Chen, Karen A. Robinson, Joel J. Gagnier, Stephan Ehrhardt, David D. Celentano

**Affiliations:** 1 Bloomberg School of Public Health, The Johns Hopkins University, Baltimore, Maryland; 2 Welch Medical Library, The Johns Hopkins University, Baltimore, Maryland; 3 Institute of Information and Medical Library, Peking Union Medical College and Chinese Academy of Medical Sciences, Beijing, China; 4 Department of Medicine, School of Medicine, The Johns Hopkins University, Baltimore, Maryland; 5 Michigan Medicine, School of Public Health, University of Michigan, Ann Arbor

## Abstract

**Importance:**

Language and indexing biases may exist among Chinese-sponsored randomized clinical trials (CS-RCTs). Such biases may threaten the validity of systematic reviews.

**Objective:**

To evaluate the existence of language and indexing biases among CS-RCTs on drug interventions.

**Design, Setting, and Participants:**

In this retrospective cohort study, eligible CS-RCTs were retrieved from trial registries, and bibliographic databases were searched to determine their publication status. Eligible CS-RCTs were for drug interventions conducted from January 1, 2008, to December 31, 2014. The search and analysis were conducted from March 1 to August 31, 2019. Primary trial registries were recognized by the World Health Organization and the Drug Clinical Trial Registry Platform sponsored by the China Food and Drug Administration.

**Exposures:**

Individual CS-RCTs with positive vs negative results (positive vs negative CS-RCTs).

**Main Outcomes and Measures:**

For assessing language bias, the main outcome was the language of the journal in which CS-RCTs were published (English vs Chinese). For indexing bias, the main outcome was the language of the bibliographic database where the CS-RCTs were indexed (English vs Chinese).

**Results:**

The search identified 891 eligible CS-RCTs. Four hundred seventy CS-RCTs were published by August 31, 2019, of which 368 (78.3%) were published in English. Among CS-RCTs registered in the Chinese Clinical Trial Registry (ChiCTR), positive CS-RCTs were 3.92 (95% CI, 2.20-7.00) times more likely to be published in English than negative CS-RCTs; among CS-RCTs in English-language registries, positive CS-RCTs were 3.22 (95% CI, 1.34-7.78) times more likely to be published in English than negative CS-RCTs. These findings suggest the existence of language bias. Among CS-RCTs registered in ChiCTR, positive CS-RCTs were 2.89 (95% CI, 1.55-5.40) times more likely to be indexed in English bibliographic databases than negative CS-RCTs; among CS-RCTs in English-language registries, positive CS-RCTs were 2.19 (95% CI, 0.82-5.82) times more likely to be indexed in English bibliographic databases than negative CS-RCTs. These findings support the existence of indexing bias.

**Conclusions and Relevance:**

This study suggests the existence of language and indexing biases among registered CS-RCTs on drug interventions. These biases may distort evidence synthesis toward more positive results of drug interventions.

## Key Points

**Question:**

Do language and indexing biases exist among Chinese-sponsored randomized clinical trials on drug interventions?

**Findings:**

This cohort study included 891 eligible Chinese-sponsored randomized clinical trials identified from English- and Chinese-language clinical trial registries. Among 470 published trials as of August 2019, positive trial findings were more commonly published in English-language journals and indexed in English-language bibliographic databases than negative trial findings.

**Meaning:**

These findings suggest that language and indexing biases may lead to distorted, more positive results of drug interventions when synthesizing evidence.

## Teaser

This cohort study evaluates the existence of language and indexing biases among Chinese-sponsored randomized clinical trials on drug interventions.

## Introduction

In non–English-speaking countries, researchers can choose to publish randomized clinical trials (RCTs) in English-language journals or journals in their native language. It is established that RCTs with positive results (hereinafter referred to as positive RCTs) are more likely to be published in English-language journals, a phenomenon termed *language bias*.^1^ This tendency may lead to disproportionally more positive RCTs in English literature and consequently more RCTs with negative findings (hereinafter referred to as negative RCTs) in non-English literature.^2^

Ideally, this bias would not threaten the validity of systematic reviews because reviewers should comprehensively search for all existing evidence regardless of the language^1^; however, estimates indicate that almost 40% of systematic reviews were reportedly restricted to English-language articles indexed in English bibliographic databases.^3^ This estimate raises the concern that such reviews may miss negative RCTs that are only published in the non-English literature, leading to biased evidence.^4^

Recently, scientific publications from Mainland China have been surging.^5^ Publications of RCTs sponsored by researchers in Mainland China (Chinese-sponsored RCTs [CS-RCTs]) are also split between English- and Chinese-language journals (English CS-RCTs and Chinese CS-RCTs).^6^ However, limited evidence is available regarding language bias among CS-RCTs. Whether we should include Chinese CS-RCTs indexed in English bibliographic databases to reduce the effect of speculated language bias is unknown.

A further challenge, and one more difficult to address, is that most Chinese journals have not been indexed in English bibliographic databases owing to large quantity and varying quality.^7^ This challenge implies that systematic reviewers must not only remove language restrictions from searching English bibliographic databases but also actively search Chinese bibliographic databases to capture all Chinese CS-RCTs, a practice seldomly adopted by the systematic review community.^8^ Bias may exist if Chinese CS-RCTs with positive results are more likely to be indexed in English bibliographic databases than their negative counterparts, which are more commonly seen in Chinese bibliographic databases. We term this potential residual of language bias as *indexing bias*.

Currently, the *Cochrane Handbook for Systematic Reviews of Interventions *only recommends searching Chinese bibliographic databases for systematic reviews on Chinese herbal medicine.^1^ Whether the recommendation should be extended to drug interventions is unknown. The objective of this study was to evaluate the existence of language and indexing biases among CS-RCTs on drug interventions to inform the potential update of the recommendation.

## Methods

In this retrospective cohort study, we retrieved CS-RCTs from trial registries and searched bibliographic databases to determine their publication status. Two hypotheses were predefined: (1) positive CS-RCTs were more likely to be published in English than negative CS-RCTs (language bias), and (2) positive CS-RCTs were more likely to be indexed in English than Chinese bibliographic databases (indexing bias). Because this study was a literature review based on open-source data and did not include research participants, it was not subject to institutional review board approval. We followed the Strengthening the Reporting of Observational Studies in Epidemiology (STROBE) reporting guideline.^9^

### Identifying CS-RCTs From Trial Registries

We retrieved CS-RCTs from all 17 primary registries recognized by the World Health Organization^10^ and the Drug Clinical Trial Registry Platform (DCTRP) sponsored by the China Food and Drug Administration.^11^ A substance was considered a drug if recognized and regulated by the US Food and Drug Administration and/or the European Medicines Agency.^12,13^ We included all CS-RCTs that started January 1, 2008, and were completed by December 31, 2014, to allow a minimum of 4.5 years from trial completion to publication.^14^ We excluded phase 1 trials (including bioequivalence and pharmacokinetics studies) and CS-RCTs missing the study period or RCTs with an unclear study interval (eg, end date before the start date) or any unnamed experimental drug, principal investigator, or sponsor in the registries.

### Identification of Journal Articles From Bibliographic Databases

The search and analysis were conducted from March 1 to August 31, 2019. We only included journal articles produced from eligible CS-RCTs. Conference abstracts, research letters, and dissertations were not included. Publications of protocols, subgroup analyses, secondary analyses, and meta-analyses were also excluded.

Based on previous studies,^14,15^ we developed search strategies for individual CS-RCTs with informationists from the Welch Medical Library at The Johns Hopkins University (L.R.) and the Institute of Information and Medical Library at Peking Union Medical College (Q.C.) (eTable 1 in the Supplement).^16,17^ We expanded the search terms with synonyms and spelling variations to increase sensitivity. The search terms were tailored and organized for each bibliographic database based on the database’s specific syntax (eTable 2 in the Supplement). Seven bibliographic databases, including 3 English language (PubMed, Embase, and the Cochrane Central Register of Controlled Trials [CENTRAL]) and 4 Chinese language (China National Knowledge Infrastructure, SinoMed, VIP information, and Wanfang Data), were subsequentially searched.^18^

We conducted a 4-step process to identify matches of eligible CS-RCTs. First, we searched bibliographic databases to retrieve citations; second, we screened the citations for eligible CS-RCTs; third, we downloaded PDFs of possibly eligible trials; and fourth, we matched the PDFs with the registry records of eligible CS-RCTs. The criteria for screening and matching are shown in eTable 3 in the Supplement.

The journal articles were classified as confirmed matches and probable matches according to the similarity between journal articles and registry records. Confirmed matches indicated the journal articles were consistent with the registry records, whereas probable matches indicated the journal articles were similar to the registry records but differed on or lacked only 1 data item. The primary analysis was conducted among the confirmed and probable matches, whereas a sensitivity analysis was conducted among the confirmed matches only.

Two authors (Y.J., D.H.) independently searched bibliographic databases and identified matching PDFs of eligible CS-RCTs. Discrepancies were discussed and resolved by a third author (J.W.). If multiple journal articles existed, we only considered the one with the largest sample size or the earliest one if identical sample size was reported in multiple articles.

### Statistical Analysis

#### Exposure

The exposure was the finding of individual CS-RCTs (positive vs negative) according to the CS-RCT’s primary outcome reported in the journal article.^19^ If multiple primary outcomes were reported in a CS-RCT, we selected the first one reported in the result section. If no primary outcome was defined, the selection of the CS-RCT’s primary outcome was based on the following hierarchical order: the first outcome used in the sample size calculation, the first outcome defined in the study objective, or the first outcome reported in the Results section. When the time point was not specified for the CS-RCT’s primary outcome that was measured at multiple time points, we considered the last point in our main analysis and the first point in sensitivity analyses.

We defined a positive result as favoring the experimental group with statistical significance in superiority trials or showing no difference between treatment groups for equivalence or noninferiority trials. Results that were not statistically significant, significantly favored the control group, or failed to show equivalence or noninferiority were defined as negative.

#### Outcome

Two main outcomes were defined: the language of the publication (English vs Chinese) and the language of the bibliographic database where the publication was indexed (English vs Chinese). An article published in both Chinese and English was considered published in English; similarly, an article indexed in both English and Chinese bibliographic databases was considered indexed in an English bibliographic database. We assumed all English-language articles were indexed in English bibliographic databases, but Chinese-language articles were possibly indexed in English or Chinese bibliographic databases.

#### Measurements of Associations

Bias was estimated by relative risk (RR), including point estimates and 95% CIs. An RR larger than 1.00 indicated that positive CS-RCTs were more likely than negative CS-RCTs to be published in English or indexed in English bibliographic databases. The RRs were estimated using log binomial models with 5 covariates: sample size (<100 vs ≥100), funding source (industry vs nonindustry), study design (superiority vs noninferiority or equivalence), number of recruitment centers (single vs multiple), and registration type (prospective vs retrospective).^20,21^ Industrial funding was considered as long as 1 funder was from industry; prospective registration was considered when registration occurred before the first participant was recruited.^22^ We included an interaction term in the models to evaluate the heterogeneity of bias across registries. Statistical significance was defined as 2-sided *P* < .05 for the main effect and *P* < .10 for interaction, generated by Wald χ^2^ tests as in the log binomial models. SAS, version 9.4 (SAS Institute Inc) was used for data cleaning and analysis.

## Results

The search through trial registries and bibliographic databases was conducted from March 1 to August 31, 2019. Among the 17 primary registries and DCTRP, 5 were found to include eligible CS-RCTs: the Chinese Clinical Trial Registry (ChiCTR), ClinicalTrials.gov, International Standard Randomized Controlled Trials Number (ISRCTN), the Australia New Zealand Clinical Trials Registry (ANZCTR), and DCTRP. In total, 5084 CS-RCTs were retrieved from these trial registries and screened for eligibility. Eventually, 891 eligible CS-RCTs were identified, 470 (52.8%) of which had been published in 229 English-language journals and 72 Chinese-language journals. The screening results are shown in the Figure.

**Figure.**
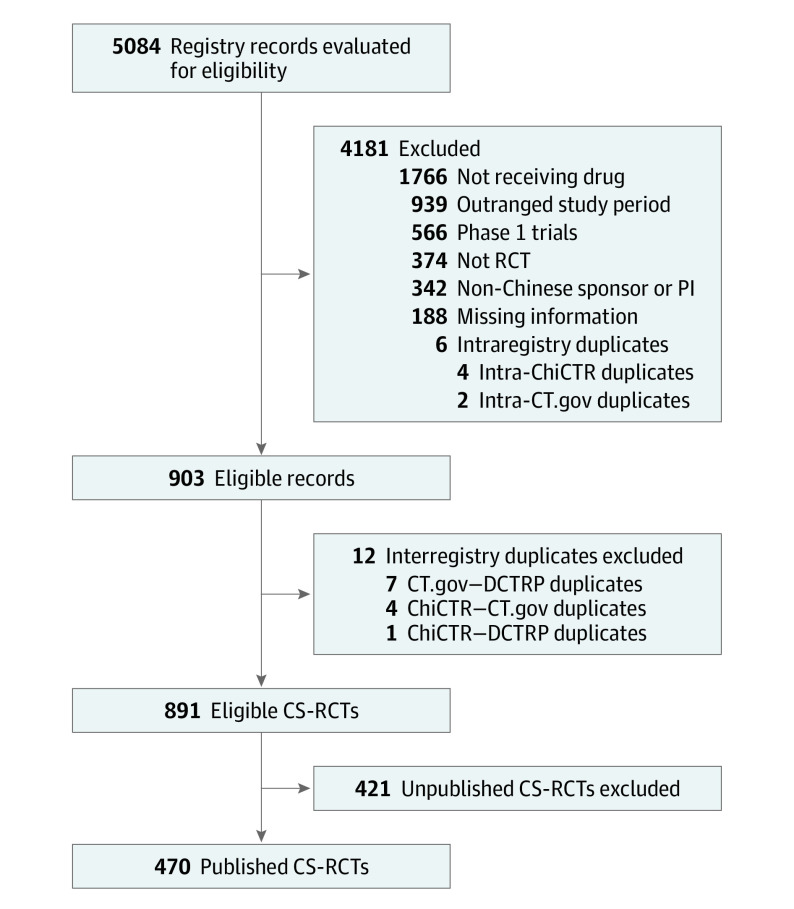
Study Flowchart Identifying Published Chinese-Sponsored Randomized Clinical Trials (CS-RCTs) ChiCTR indicates Chinese Clinical Trial Registry; CT.gov, ClinicalTrials.gov; DCTRP, Drug Clinical Trial Registry Platform; and PI, principal investigator.

### Characteristics of CS-RCTs

Among the 470 journal articles corresponding to 470 CS-RCTs, 368 (78.3%) were published in English and 102 (21.7%) were in Chinese; 432 (91.9%) were confirmed matches to registry records and 38 (8.1%) were probable matches. Thirty of 38 probable matches (79.0%) were published in Chinese. The matching results are shown in eTable 3 in the Supplement.

The distribution of CS-RCTs across bibliographic databases is shown in Table 1. The 3 English bibliographic databases only indexed a small proportion of Chinese articles, ranging from 22 of 102 (21.6%) by PubMed to 24 of 102 each (23.5%) by Embase and CENTRAL. The low coverage of the China National Knowledge Infrastructure was mainly due to unindexed or partially unindexed medical journals sponsored by the Chinese Medical Association.

**Table 1.** Coverage of Journal Articles by Bibliographic Databases

Bibliographic database	Article language, No. (%)					
	English (n = 368)		Chinese (n = 102)		All (N = 470)	
	Indexed	Unindexed	Indexed	Unindexed	Indexed	Unindexed
English bibliographic databases						
PubMed	357 (97.0)	11 (3.0)	22 (21.6)	80 (78.4)	379 (80.6)	91 (19.4)
Embase	364 (98.9)	4 (1.1)	24 (23.5)	78 (76.5)	388 (82.6)	82 (17.4)
CENTRAL	348 (94.6)	20 (5.4)	24 (23.5)	78 (76.5)	372 (79.1)	98 (20.9)
Chinese bibliographic databases						
SinoMed	NA	NA	102 (100)	0	NA	NA
CNKI	NA	NA	66 (64.7)	36 (35.3)	NA	NA
VIP Data	NA	NA	100 (98.0)	2 (2.0)	NA	NA
Wanfang Data	NA	NA	101 (99.0)	1 (1.0)	NA	NA

Abbreviations: CENTRAL, Cochrane Central Register of Controlled Trials; CNKI, China National Knowledge Infrastructure; NA, not applicable.

Most CS-RCTs (306 [65.1%]) were registered in ChiCTR, followed by ClinicalTrials.gov (143 [30.4%]), DCTRP (13 [2.8%]), ISRCTN (4 [0.9%]), and ANZCTR (1 [0.2%]). The CS-RCTs in DCTRP, ISRCTN, and ANZCTR were too few to be analyzed separately. We expected that both biases would be similar across ClinicalTrials.gov, ISRCTN, and ANZCTR, so we combined CS-RCTs from these 3 registries to form an English registry category. On the other hand, DCTRP, which was only available in Chinese and was not a primary registry, was excluded from the inferential analyses. Of the 470 CS-RCTs, 323 (68.7%) were positive, 2 (0.4%) were excluded from inferential analyses because of unknown positivity (no intergroup comparison), 286 (60.9%) recruited at least 100 participants, 322 (68.5%) were conducted at a single center, 377 (80.2%) were supported by nonindustry funding, 315 (67.0%) were retrospectively registered, and 442 (94.0%) were superiority trials.

The distribution of covariates, including the sample size, funding source, study design, number of recruitment centers, and registration type, was similar between English and Chinese CS-RCTs (eTable 4 in the Supplement). The distribution of covariates among CS-RCTs indexed in English bibliographic databases was similar to that of CS-RCTs only indexed in Chinese bibliographic databases, although the number of positive CS-RCTs indexed in Chinese bibliographic databases was slightly larger than the number of negative CS-RCTs (eTable 5 in the Supplement).

### Language Bias

Four hundred sixty-eight CS-RCTs were included for this analysis. As shown in Table 2, positive CS-RCTs were more likely to be published in English (180 of 211 [85.3%] vs 31 of 211 [14.7%]) and were more likely to be indexed in English bibliographic databases (186 of 211 [88.2%] vs 25 of 211 [11.8%]) compared with CS-RCTs published in Chinese. After adjusting for covariates, positive CS-RCTs were more commonly published in English than negative CS-RCTs. The RRs were 3.92 (95% CI, 2.20-7.00) and 3.22 (95% CI, 1.34-7.78) among CS-RCTs registered in ChiCTR or English-language registries, respectively (Table 3). The interaction between registry and positivity of CS-RCTs was not statistically significant (Wald χ^2^ = 0.13; *P* = .71), indicating no evidence of heterogeneity of language bias across registries. Other factors associated with increased likelihood of being published in English among CS-RCTs were a sample size of at least 100 (RR, 2.09; 95% CI, 1.19-3.67), single-center as opposed to multicenter trials (RR, 1.85; 95% CI, 1.01-3.41), and financial support from a nonindustrial source compared with an industrial source (RR, 1.99; 95% CI, 1.06-3.75).

**Table 2.** Positivity of CS-RCTs by Trial Registry and Bibliographic Database

Trial registry by RCT finding	Article language, No. (%)		Bibliographic database language, No. (%)	
	English	Chinese	English	Chinese
**ChiCTR**				
Positive	180 (85.3)	31 (14.7)	186 (88.2)	25 (11.8)
Negative	56 (59.6)	38 (40.4)	67 (71.3)	27 (28.7)
All	236 (77.4)	69 (22.6)	253 (83.0)	52 (17.0)
**English-language registries**				
Positive	91 (87.5)	13 (12.5)	93 (89.4)	11 (10.6)
Negative	32 (69.6)	14 (30.4)	37 (80.4)	9 (19.6)
All	123 (82.0)	27 (18.0)	130 (86.7)	20 (13.3)
**DCTRP**				
Positive	5 (62.5)	3 (37.5)	6 (75.0)	2 (25.0)
Negative	2 (40.0)	3 (60.0)	4 (80.0)	1 (20.0)
All	7 (53.8)	6 (46.3)	10 (76.9)	3 (23.1)
**Total**				
Positive	276 (85.4)	47 (14.6)	285 (88.2)	38 (11.8)
Negative	90 (62.1)	55 (37.9)	108 (74.5)	37 (25.5)
All	366 (78.2)	102 (21.8)	393 (84.0)	75 (16.0)

Abbreviations: ChiCTR, Chinese Clinical Trial Registry; CS-RCT, Chinese-sponsored randomized clinical trial; DCTRP, Drug Clinical Trial Registry Platform.

**Table 3.** Factors Associated With Language Bias and Indexing Bias

Factor	Language bias		Indexing bias	
	RR (95% CI)	*P* value	RR (95% CI)	*P* value
Findings				
ChiCTR				
Positive	3.92 (2.20-7.00)	<.001	2.89 (1.55-5.40)	.001
Negative	1 [Reference]	NA	1 [Reference]	NA
English-language registries				
Positive	3.22 (1.34-7.78)	.009	2.19 (0.82-5.82)	.12
Negative	1 [Reference]	NA	1 [Reference]	NA
Sample size				
≥100	2.09 (1.19-3.67)	.01	2.04 (1.11-3.72)	.02
<100	1 [Reference]	NA	1 [Reference]	NA
No. of centers				
Single	1.85 (1.01-3.41)	.049	1.56 (0.80-3.05)	.20
Multiple	1 [Reference]	NA	1 [Reference]	NA
Funding				
Nonindustry	1.99 (1.06-3.75)	.03	1.29 (0.63-2.67)	.48
Industry	1 [Reference]	NA	1 [Reference]	NA
Registration type				
Retrospective	1.43 (0.85-2.40)	.17	1.47 (0.84-2.56)	.17
Prospective	1 [Reference]	NA	1 [Reference]	NA
Design				
Superiority	1.17 (0.36-3.80)	.80	1.30 (0.35-4.78)	.70
Equivalence or noninferiority	1 [Reference]	NA	1 [Reference]	NA

Abbreviations: ChiCTR, Chinese Clinical Trial Registry; NA, not applicable; RR, relative risk.

### Indexing Bias

Four hundred sixty-eight CS-RCTs were included for this analysis. As shown in Table 2, English-language CS-RCTs (91 of 104 [87.5%] vs 13 of 104 [12.5%]) or CS-RCTs indexed in English bibliographic databases (93 of 104 [89.4%] vs 11 of 104 [10.6%]) were more likely to be positive than Chinese-language CS-RCTs or CS-RCTs only indexed in Chinese bibliographic databases. After adjusting for covariates, positive CS-RCTs were more commonly indexed in English bibliographic databases than negative CS-RCTs. The RRs were 2.89 (95% CI, 1.55-5.40) and 2.19 (95% CI, 0.82-5.82) among CS-RCTs registered in ChiCTR or English-language registries, respectively (Table 3). The interaction between registry and language of bibliographic databases was not statistically significant (Wald χ^2^ = 0.22; *P* = .64), indicating no evidence of heterogeneity of indexing bias across registries. The only other factor associated with an increased likelihood of being indexed in English bibliographic databases among CS-RCTs was sample size of at least 100 (RR, 2.04; 95% CI, 1.11-3.72).

### Sensitivity Analyses

Two sensitivity analyses were conducted. When only confirmed matches (n = 432) were analyzed, the RRs increased regarding language bias (4.14 [95% CI, 2.09-8.21] among ChiCTR and 3.58 [95% CI, 1.32-9.72] among English-language registries) and decreased regarding indexing bias (2.47 [95% CI, 1.15-5.34] among ChiCTR and 1.92 [95% CI, 0.62-6.04] among English-language registries). When the first assessment of the CS-RCTs’ primary outcomes was analyzed (rather than the last assessment at the end of follow-up), the RRs increased regarding both language bias (5.34 [95% CI, 3.00-9.68] among ChiCTR and 3.73 [95% CI, 1.53-9.07] among English-language registries) and indexing bias (4.76 [95% CI, 2.49-9.08] among ChiCTR and 2.67 [95% CI, 1.01-7.11] among English-language registries).

## Discussion

Our study supports the existence of language bias and indexing bias among CS-RCTs included in trial registries. As hypothesized, positive CS-RCTs were more likely to be published in English or indexed in English bibliographic databases compared with negative CS-RCTs.

### Language Bias

Reputation, job prospects, and academic progress may critically depend on publishing in English-language journals among Chinese researchers.^23,24^ Positive CS-RCTs are more likely to be submitted to English-language journals because they typically have a higher chance of being accepted; accordingly, English-language journals contain more positive CS-RCTs than their Chinese counterparts.

Theoretically, language bias disappears if all clinical trials shift to be published in English. This ideal has been echoed by a trend toward publishing in English in some countries, such as Germany.^25^ With the mean number of RCTs per German-language journal decreasing from a maximum of 11.2 annually from 1970 to 1986 to only 1.7 annually from 2002 to 2004, language bias from German-speaking countries may no longer be a concern.

We did not detect such a trend among CS-RCTs. According to an ongoing study, the number of CS-RCTs published in Chinese may be as many as 44 000 in 2016 as opposed to fewer than 1000 being published in English (Y.J., Jun Liang, J.W., et al; unpublished data; June 2020). The deep gap between the Chinese and English literature has allowed significant space for language bias to develop.

Several studies attempted to evaluate the effect of language bias based on non–English-language trials included in systematic reviews.^26,27^ Because most systematic reviews were constrained to work within English bibliographic databases only, what those studies measured was a fraction of language bias—the difference between English-language and non–English-language trials indexed in English bibliographic databases. The effect of language bias cannot be comprehensively evaluated unless non–English-language trials, especially the ones not indexed in English bibliographic databases, are included and evaluated.

### Indexing Bias

As the primary source for systematic reviewers, English bibliographic databases may index some non–English-language literature, but they vary in the amount and scope. The *Cochrane Handbook for Systematic Reviews* and the United States Institute of Medicine Guidelines for Systematic Reviews have recommended including non–English-language literature indexed in English bibliographic databases.^1,28^ Including non–English-language trials indexed in English bibliographic databases may not eliminate the effect of language bias but could reduce it to the scope of indexing bias.

To date, English bibliographic databases do not represent the Chinese-language literature. This is problematic because an ongoing study shows more than 10 000 clinical trials have been published out of China in 2016 (Y.J., Jun Liang, J.W., et al; unpublished data; June 2020). However, Embase only indexes 80 Chinese-language journals.^7^ Although we did not simulate actual systematic reviews, it appears plausible that, owing to language and indexing bias, drug interventions might appear more positive than they are when existing evidence is synthesized, for example in systematic reviews.

### How to Eliminate the Effect of Language Bias Regarding CS-RCTs

The effect of language bias regarding CS-RCTs might be eliminated if reviews comprehensively searched Chinese bibliographic databases, if major English bibliographic databases would index all Chinese-language literature, or if all CS-RCTs would be appropriately registered with results. There are, however, layers of complexity that warrant appreciation. There has been a discussion as to whether scientists should search Chinese bibliographic databases when conducting systematic reviews.^8,29^ Our study tipped the scales in this proposition’s direction: including Chinese-language literature may reduce bias and shrink the CIs of the estimates. Currently, the *Cochrane Handbook for Systematic Reviews of Interventions* only recommends searching Chinese bibliographic databases for topics related to complementary medicine or Chinese medicine,^1^ but our results suggest that it might be prudent to expand recommendations to studies on drug interventions as well.

The reporting quality of Chinese CS-RCTs was low,^6,30,31^ which some may argue is a reason to not use Chinese CS-RCTs in systematic reviews. However, reporting quality may not completely represent the actual scientific quality.^32^ One study found no difference between one systematic review mainly using English-language trials and another mainly using Chinese-language trials on the same topic.^8^ It is the researchers’ decision to include or not include those trials (ie, trials published in Chinese and/or indexed in Chinese bibliographic databases) based on reporting quality, but it might be too simplistic to just ignore them.

### Limitations

Our study has several limitations. First, we searched 7 prominent, but not all, bibliographic databases.^33^ Second, our search strategy relied on the information in trial registries, which may be inaccurate and/or incomplete.^34,35^ Third, less than 15% of Chinese-language articles reported registration,^6^ indicating CS-RCTs in trial registries may not be representative of all CS-RCTs in the period addressed. At this moment, it is unclear how much this study can be generalized to all CS-RCTs. In addition, we studied language bias and indexing bias from the level of the entire RCT community, but we did not assess whether such biases might have effects on the conclusion of individual systematic reviews. Such simulation or empirical studies might further elucidate the extent and direction of systematic error introduced by language and indexing bias.

## Conclusions

Our study indicates the existence of language bias and indexing bias among CS-RCTs in trial registries. These biases might threaten the validity of evidence synthesis. When synthesizing evidence, drug interventions might appear more favorable than in reality owing to language and indexing bias. Removing language restrictions and actively searching Chinese bibliographic databases may reduce the effect of these 2 biases.

## Notes

Supplement. **eTable 1.** Search Strategies **eTable 2.** Search Terms **eTable 3.** Method to Match Journal Articles With Registry Records **eTable 4.** Characteristics of Published CS-RCTs by Language of Journal Articles **eTable 5.** Characteristics of Published CS-RCTs by Language of Bibliographic Databases
