# Supplementary material for: Assessment of Language and Indexing Biases Among Chinese-Sponsored Randomized Clinical Trials
Source: JAMA Netw Open. 2020 May 28;3(5):e205894. doi: 10.1001/jamanetworkopen.2020.5894 (PMC7256669; doi:10.1001/jamanetworkopen.2020.5894)
Supplement: Supplement. — eTable 1. Search Strategies eTable 2. Search Terms eTable 3. Method to Match Journal Articles With Registry Records eTable 4. Characteristics of Published CS-RCTs by Language of Journal Articles eTable 5. Characteristics of Published CS-RCTs by Language of Bibliographic Databases [file jamanetwopen-3-e205894-s001.pdf]

Supplementary Online Content

Jia Y, Huang D, Wen J, et al. Assessment of language and indexing biases among Chinese-sponsored randomized clinical trials. *JAMA Netw Open*. 2020;3(5):e205894. doi:10.1001/jamanetworkopen.2020.5894

eTable 1. Search Strategies

eTable 2. Search Terms

eTable 3. Method to Match Journal Articles With Registry Records

eTable 4. Characteristics of Published CS-RCTs by Language of Journal Articles

eTable 5. Characteristics of Published CS-RCTs by Language of Bibliographic Databases

This supplementary material has been provided by the authors to give readers additional information about their work.

©2020 Jia Y et al. *JAMA Network Open*.

eTable 1. Search Strategies

Strategy 1	Single-Center and Multi-Center CS-RCTs	Registration Number
Strategy 2	Single-Center and Multi-Center CS-RCTs	PI's Name AND PI's Affiliation AND (Disease OR Drug) AND Study End Date
Strategy 3	Multi-Center CS-RCTs	Recruitment Facilities AND Disease AND Drug AND Study End Date

PI: Principle Investigator; CS-RCT: Chinese-Sponsored Randomized Controlled Trials.

©2020 Jia Y et al. *JAMA Network Open*.

eTable 2. Search Terms

Database	Identifier	Source of Subjects	Source of Keywords
English	Disease	MeSH<br>Emtree	Registry records<br>PubMed entry terms<br>Embase synonyms
	Drug	MeSH<br>Emtree	Registry records<br>PubMed entry terms<br>Embase synonyms
Chinese	Disease	MeSH (Chinese version)	Registry records<br>ICD-10 (Chinese version)<br>ICD-9 (Chinese version)<br>Three doctors from PUMC
	Drug	MeSH (Chinese version)	Registry records<br>China FDA website for drug trade names and compound names<br>Three pharmacists from PUMC

MeSH: Medical Subject Headings; Emtree: Embase Subject Headings; ICD-9: The International Classification of Diseases, Ninth Revision; ICD-10: The International Classification of Diseases, Tenth Revision; PUMC: Peking Union Medical College; China FDA: China Food and Drug Administration

©2020 Jia Y et al. *JAMA Network Open*.

eTable 3. Method to Match Journal Articles With Registry Records

Category	English Articles			Chinese Articles			Total	
	No.	%		No.	%		No.	%
Confirmed Matches								
Similar Eligibility Criteria & Interventions, Same Registration Number	311	84.5		20	19.6		331	70.4
Similar Eligibility Criteria & Interventions, Same Ethics Committee Approval Number	1	0.3		8	7.8		9	1.9
Similar Eligibility Criteria & Interventions, Same Funding Identification	2	0.5		1	1.0		3	0.6
Consistent Eligible Criteria & Interventions, Similar Sample Size & Overlapped Study Period	38	10.3		40	39.2		78	16.6
Consistent Eligible Criteria & Interventions, Identical Sample Size	7	1.9		3	2.9		10	2.1
Consistent Eligible Criteria & Interventions, Identical Study Period	1	0.3		0	0.0		1	0.2
Total	360	97.8		72	70.6		432	91.9
Probable Matches								
Consistent Eligibility Criteria & Interventions Similar Sample Size	7	1.9		16	15.7		23	4.9
Consistent Eligibility Criteria & Interventions Overlapped Study Period	1	0.3		14	13.7		15	3.2
Total	8	2.2		30	27.4		38	8.1
Total	368	100.0		102	100.0		470	100.0

©2020 Jia Y et al. *JAMA Network Open*.

eTable 4. Characteristics of Published CS-RCTs by Language of Journal Articles

**Category**	**English Articles**				**Chinese Articles**				**Total**		
	**No.**		**%**		**No.**		**%**		**No.**		**%**
Positivity											
Positive	276		75.41		47		46.1		323		69.0
Negative	90		24.59		55		53.9		145		31.0
Sample Size											
>=100	230		62.5		56		54.9		286		60.9
<100	138		37.5		46		45.1		184		39.1
Number of Centers											
Multi-center	108		29.35		40		39.2		148		31.5
Single-center	260		70.65		62		60.8		322		69.5
Funding											
Industry	63		17.12		30		29.4		93		19.8
Non-Industry	305		82.88		72		70.6		377		80.2
Registration Type											
Prospective	115		31.25		40		39.2		155		33.0
Retrospective	253		68.75		62		60.8		315		67.0
Design											
Superiority	346		94.54		96		94.1		442		94.4
Equivalence or Non-inferiority	20		5.46		6		5.9		26		5.6
Total	368		100.0		102		100.0		470		100.0

Abbreviations

CS-RCTs: Chinese-Sponsored Randomized Controlled Trials.

©2020 Jia Y et al. *JAMA Network Open*.

eTable 5. Characteristics of Published CS-RCTs by Language of Bibliographic Databases

**Category**	**English Databases**		**Chinese Databases**		**Total**	
	**No.**	**%**	**No.**	**%**	**No.**	**%**
Positivity						
Positive	285	72.5	38	50.7	323	69.0
Negative	108	27.5	37	49.3	145	31.0
Sample Size						
<100	147	37.2	37	49.3	184	39.2
>=100	248	62.8	38	50.7	286	60.8
Number of Centers						
Multi-center	123	31.1	25	33.3	148	31.5
Single-center	272	68.9	50	66.7	322	68.5
Funding						
Industry	76	19.2	17	22.7	93	19.8
Non-Industry	319	80.8	58	77.3	377	80.2
Registration Type						
Prospective	125	31.7	30	40.0	155	33.0
Retrospective	270	68.3	45	60.0	315	67.0
Design						
Superiority	371	94.4	71	94.7	442	94.4
Equivalence or Non-inferiority	22	5.6	4	5.3	26	5.6
Total	395	100.0	75	100.0	470	100.0

Abbreviations

CS-RCTs: Chinese-Sponsored Randomized Controlled Trials.

©2020 Jia Y et al. *JAMA Network Open*.
